# Simplified meal announcement study (SMASH) using hybrid closed-loop insulin delivery in youth and young adults with type 1 diabetes: a randomised controlled two-centre crossover trial

**DOI:** 10.1007/s00125-024-06319-w

**Published:** 2024-11-19

**Authors:** Céline I. Laesser, Camillo Piazza, Nina Schorno, Fabian Nick, Lum Kastrati, Thomas Zueger, Katharine Barnard-Kelly, Malgorzata E. Wilinska, Christos T. Nakas, Roman Hovorka, David Herzig, Daniel Konrad, Lia Bally

**Affiliations:** 1 Division of Paediatric Endocrinology and Diabetology, University Children’s Hospital, University of Zurich, Zurich, Switzerland; 2 Children’s Research Centre, University Children’s Hospital, University of Zurich, Zurich, Switzerland; 3 Department of Diabetes, Endocrinology, Nutritional Medicine and Metabolism UDEM, Inselspital, Bern University Hospital, University of Bern, Bern, Switzerland; 4 Institute of Social and Preventive Medicine (ISPM), University of Bern, Bern, Switzerland; 5 Graduate School for Health Sciences, University of Bern, Bern, Switzerland; 6 Department of Endocrinology and Metabolic Diseases, Kantonsspital Olten, Olten, Switzerland; 7 Southern Health NHS Foundation Trust, Southampton, UK; 8 BHR Limited, Portsmouth, Hampshire UK; 9 Institute of Metabolic Science, University of Cambridge, Cambridge, UK; 10 School of Agricultural Sciences, University of Thessaly, Laboratory of Biometry, Volos, Greece; 11 Department of Clinical Chemistry, Inselspital, Bern University Hospital, University of Bern, Bern, Switzerland

**Keywords:** Devices, Diabetes in childhood, Nutrition and diet

## Abstract

**Aims/hypothesis:**

The majority of hybrid closed-loop systems still require carbohydrate counting (CC) but the evidence for its justification remains limited. Here, we evaluated glucose control with simplified meal announcement (SMA) vs CC in youth and young adults with type 1 diabetes using the mylife CamAPS FX system.

**Methods:**

We conducted a two-centre, randomised crossover, non-inferiority trial in two University Hospitals in Switzerland in 46 participants (aged 12–20 years) with type 1 diabetes using multiple daily injections (*n*=35), sensor-augmented pump (*n*=4) or hybrid closed-loop (*n*=7) therapy before enrolment. Participants underwent two 3 month periods with the mylife CamAPS FX system (YpsoPump, Dexcom G6) to compare SMA (individualised carbohydrate meal sizes) with CC, in a randomly assigned order using computer-generated sequences. The primary endpoint was the proportion of time glucose was in target range (3.9–10.0 mmol/l) with a non-inferiority margin of 5 percentage points. Secondary endpoints were other sensor glucose and insulin metrics, usability and safety endpoints.

**Results:**

Forty-three participants (18 women and girls) completed the trial. In the intention-to-treat analysis, time in range (mean±SD) was 69.9±12.4% with SMA and 70.7±13.0% with CC (estimated mean difference −0.6 percentage points [95% CI −2.4, 1.1], demonstrating non-inferiority). Time <3.9 mmol/l (median [IQR] 1.8 [1.2–2.2]% vs 1.9 [1.6–2.5]%) and >10.0 mmol/l (28.2±12.6% vs 27.2±13.4%) was similar between periods. Total daily insulin dose was higher with SMA (54.0±14.7 U vs 51.7±12.1 U, *p*=0.037). Three participants experienced serious adverse events, none of which were intervention-related.

**Conclusions/interpretation:**

Glucose control using the CamAPS FX algorithm with SMA was non-inferior to its use with CC in youth and young adults with type 1 diabetes.

**Trial registration:**

ClinicalTrials.gov NCT05481034.

**Funding:**

The study was supported by the Swiss Diabetes Foundation and by a YTCR grant from the Bangerter-Rhyner Foundation and the Swiss Academy of Medical Sciences. Dexcom and Ypsomed provided product support.

**Graphical Abstract:**

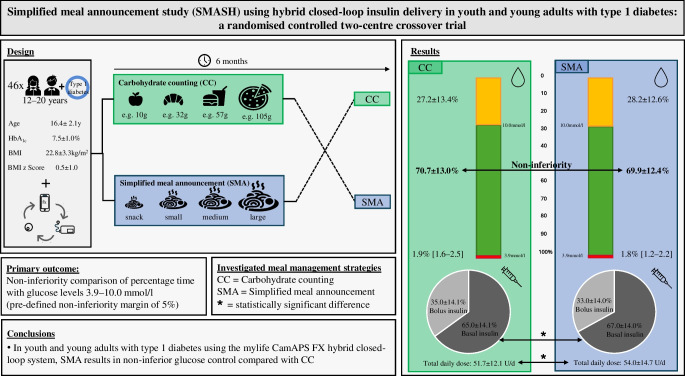

**Supplementary Information:**

The online version of this article (10.1007/s00125-024-06319-w) contains peer-reviewed but unedited supplementary material.


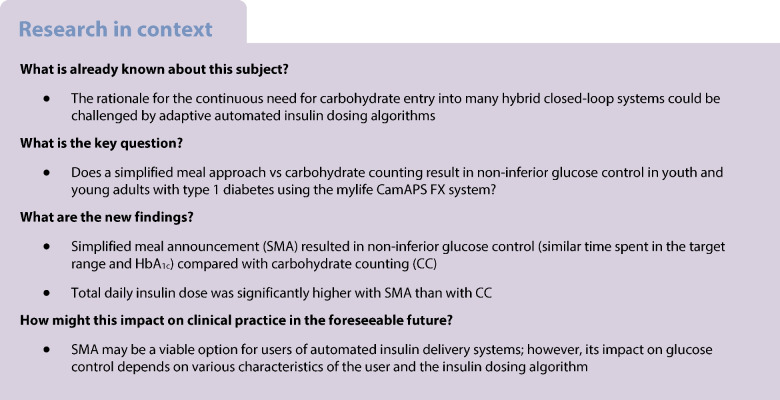


## Introduction

Hybrid closed-loop (HCL) insulin therapy, combining automated insulin dosing with user-initiated meal bolusing, is becoming the standard of care for people with type 1 diabetes in many countries [1, 2]. HCL therapy helps adult and paediatric populations with type 1 diabetes to better manage their glucose while reducing the burden of self-management [3]. However, most commercial HCL systems still require users to enter the counted number of grams of carbohydrates (CHO) in a meal for prandial insulin delivery.

While meal CHO are the main driver of postprandial glucose excursions [4], the rationale for carbohydrate counting (CC) in numbers of grams is challenged by several factors. First, the evidence supporting the efficacy of CC on glucose control is limited [5]. Second, the physiology of postprandial control is complex and dependent on carbohydrate quality, other meal constituents, gastric emptying kinetics and timing of meal insulin boluses [6–8]. Third, CC is error-prone [9] and adaptive glucose control algorithms may accommodate imprecisions in CC [10].

Regardless of its relevance for glucose control, the perceived burden of CC is high [11] and may challenge treatment engagement in youth and young adults with type 1 diabetes. With only 17% achieving recommended treatment targets [12], glucose control of youth and young adults with type 1 diabetes is worse compared with other age groups [12–14]. The required level of literacy and perceived burden of CC may confer a relevant barrier to the adoption of HCL insulin therapy [11, 15], which may be overcome by a more pragmatic meal management strategy with announcement of standard CHO meal sizes.

Thus, the objective of this trial was to compare glucose control during HCL insulin therapy with SMA (simplified meal announcement) vs CC in youth and young adults with type 1 diabetes using HCL insulin therapy.

## Methods

### Study design and participants

SMASH (simplified meal announcement study) was an open-label, two-centre, two-period (each 3 months), crossover randomised trial conducted in two tertiary care specialist diabetes centres in Switzerland (Bern and Zurich). Prior to study commencement, approval was received by the Ethics Committees in Bern and Zurich, Switzerland (BASEC-ID 2022-D0087) and the study was pre-registered with clinicaltrials.gov (NCT05481034). Study monitoring was performed by the Clinical Trials Unit (University of Bern). Participants or parents/guardians (for participants aged 12–13 years) signed informed consent before any study-related activities. Consecutive eligible patients were informed about the study and invited to participate, in an effort to obtain a study sample representative of the broader population of interest. The participants’ sex was self-reported.

Key inclusion criteria were diagnosis of type 1 diabetes for at least 6 months, age 12–20 years (inclusive), HbA_1c_ levels ≤107.7 mmol/mol (12.0%) and any prior insulin treatment modality to generate a representative sample of youth and young adults with type 1 diabetes. Key exclusion criteria were pregnancy or planned pregnancy (detailed eligibility criteria are reported in electronic supplementary material [ESM] Table 1).

### Procedures

Patients were screened for eligibility with locally measured HbA_1c_ (DCA Vantage Analyzer, Siemens Healthcare Diagnostics, Tarrytown NY, USA) and a urine pregnancy test in women and girls of childbearing age. The study flow is outlined in ESM Fig. 1. Following enrolment, participants were randomised to CC-SMA or SMA-CC using permuted block randomisation. The randomisation sequences were generated in R and uploaded into the randomisation module in REDCap by a person not involved in the study, to ensure allocation concealment until the time of randomisation. There was no washout between periods.

Before starting the first period, participants attended training and received instruction on the allocated meal management intervention. Participants were provided with study equipment and underwent measurement of HbA_1c_, weight and height. Participants filled in questionnaires for psychosocial metrics and dietary habits (food frequency questionnaire) [16, 17] and completed an electronic food picture quiz to evaluate their CC skills (16 dishes, meal CHO content 7–140 g).

After completing the first study period, participants attended the research facility before crossing over to the second period. Study assessments (HbA_1c_, anthropometrics, questionnaires) were re-measured. Meal settings in the HCL app were changed according to allocated intervention sequence, with respective instructions for use, and body weight was updated, where applicable.

At the end of the study, participants underwent the study assessments (HbA_1c_, anthropometrics, questionnaires), returned the devices and transitioned back to usual care treatments as per arrangements with their treating diabetologists.

Throughout the study, participants or their treating diabetologists were free to adjust their diabetes therapy. No active treatment optimisation, dietary restrictions or remote monitoring were undertaken by the research team.

All participants were provided with a 24 h telephone helpline to contact the research team for study-related support.

### HCL system and meal management intervention

All participants were treated with the mylife CamAPS FX system consisting of the mylife CamAPS FX app (CamDiab, Cambridge, UK) on an Android phone (study phone or compatible private phone of participants), the Dexcom G6 sensor (Dexcom, San Diego, CA, USA) and the YpsoPump (Ypsomed, Burgdorf, Switzerland) with Orbit soft or micro or YpsoPump Inset infusion sets. The HCL app used the Cambridge adaptive model predictive control algorithm to modulate pump insulin delivery every 8–12 min, according to an adjustable glucose target (4.4–11.0 mmol/l). HCL was initialised using the participant’s body weight, total daily insulin dose (TDD) and the default target of 5.8 mmol/l.

**Fig. 1:**
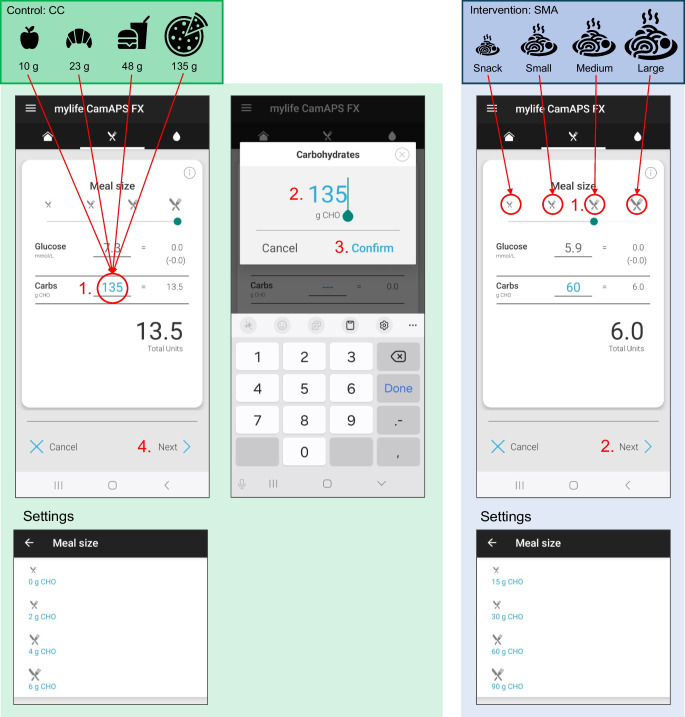
User interface of the bolus calculator on the mylife CamAPS app used for CC (left) and SMA (right). During CC, participants entered the counted number of grams of carbohydrates (red circle). To avoid using the meal size icons along the selection line at the top of the bolus calculator, standard CHO meal sizes were pre-set to minimum values (0 g, 2 g, 4 g, 6 g) in ‘Settings’ prior to starting the CC period. Before starting the SMA period, standard CHO meal sizes were personalised according to participants’ 3 day carbohydrate intake record, and participants were asked to tap on the meal size icons for prandial insulin delivery. During both periods, prandial insulin doses were calculated based on the programmed CIR

Participants were instructed to use the bolus calculator in the app for meal insulin bolus delivery (see Fig. 1), which was identical during both periods but navigated differently. In the CC period, participants were asked to enter the number of grams in CHO into the bolus calculator (lower line for CHO entry). For the SMA period, standard CHO meal sizes were set on the basis of a 3-day CHO intake record prior to starting the first study period. Participants quantified consumed CHO by means of their usual CC method and provided documentation according to their preference. The mean CHO intake per meal was rounded and classified as a medium meal size. A snack was defined as 25%, a small meal 50% and a large meal 150% thereof. In SMA, participants were asked to choose between one of the four meal size icons on the bolus calculator in the app (upper section of the bolus calculator). In the CC period, standard CHO meal sizes were set to minimum values (0 g, 2 g, 4 g, 6 g) to avoid using the meal size icons. During both periods, the bolus dose was calculated based on the programmed carbohydrate to insulin ratio (CIR).

Additionally, participants were asked not to apply advanced meal functions in the HCL app except for hypoglycaemia correction. CIR and mandatory settings were programmed during the on-boarding visit. We recommended using the system with a single CIR over a 24 h period. Changes in the settings during the course of study were recorded. Given the systems’ compatibility with both ultra-fast and fast-acting insulin analogues, participants were free to choose, but were asked to keep the type of insulin identical throughout the study.

### Endpoints

The primary endpoint assessment was a non-inferiority comparison between the percentage of time spent with glucose levels between 3.9 and 10.0 mmol/l during each study period. The non-inferiority margin was set to 5 percentage points (pp), in line with the international consensus on a clinically meaningful difference [18].

Secondary endpoints included time spent at glucose levels >10.0 mmol/l, >13.9 mmol/l, <3.9 mmol/l and <3.0 mmol/l; mean sensor glucose; glucose variability measured by CV and SD; extended hypoglycaemia and hyperglycaemia event rate (number of events with glucose <3.9 mmol/l or >13.9 mmol/l lasting for ≥120 min); number of hypoglycaemia events lasting ≥15 min (<3.0 mmol/l, <3.9 mmol/l); HbA_1c_; glucose management indicator (GMI); and peak postprandial glucose levels (assessed within 180 min following CHO entry >25 g).

Additional points of interest included insulin doses, CHO metrics, utility evaluations (per cent time of sensor glucose availability and HCL operation when sensor glucose was available) and questionnaires assessing psychosocial metrics and dietary habits. Safety evaluation included the frequency of severe hypoglycaemia, diabetic ketoacidosis and other adverse or serious adverse events (SAEs).

### Statistical analysis

Assuming a mean difference of 3pp in time spent with glucose levels between 3.9 and 10.0 mmol/l and an SD of 6.5pp, a sample size of 42 was determined to achieve a power of 80% at an alpha level of 0.025 for the primary endpoint using a non-inferiority margin of 5pp. To account for dropouts (expected dropout rate was 5%) we aimed for a target sample size of 45 participants. The SD was estimated based on the results of a clinical trial using the CamAPS FX system [19].

Primary and secondary endpoints were compared using generalised linear mixed-effects models. For endpoints derived from count data, a negative binomial distribution and a log link function was used. For all other endpoints, a linear mixed-effect model was used. For highly skewed data, a logarithmic transformation was applied, appropriate for the characteristics of the data. Models were adjusted for the period effect and accounted for within-participant correlations arising from the crossover design and for the variability across centres (period was considered as a fixed effect and participants nested within centres as a random effect). Model assumptions were evaluated using graphical methods including Residuals vs Fitted Values and Scale-Location Plots to assess the homoscedasticity and linearity assumptions; Normal Q-Q Plot to examine normality distribution of model residuals; Cook's Distance Plot and Residuals vs Leverage Plots to identify influential data points and detect any observations with high leverage and large residuals. Missing data were not imputed. Analyses were performed on an intention-to-treat base. For the non-inferiority comparison of the primary endpoint, SMA was considered non-inferior to CC if the lower limit of the 95% CI for the between-group difference was above the non-inferiority margin (−5pp). This is equivalent to one-sided non-inferiority testing with an alpha level of 0.025. For secondary endpoints, analysed according to a superiority framework, significance tests were based on two-sided tests with an alpha level of 0.05. In addition to intention-to-treat, we conducted a per-protocol analysis. Definitions for corresponding protocol deviation with observed frequencies and results of the per-protocol analysis are reported in ESM Tables 2 and 3. Results reported in the main manuscript refer to the intention-to-treat analysis. Data are presented as mean±SD or median [25th percentile; 75th percentile], unless stated differently. Analyses were conducted with R version 4.3.0 (The R Foundation for Statistical Computing, Vienna, Austria).

## Results

Between 17 December 2022 and 29 September 2023, we screened and contacted 76 individuals (ESM Fig. 2). One person did not meet the inclusion criteria, and 29 contacted people declined to participate. Forty-six participants were included in the study (deviation from target sample of 45 due to simultaneous recruitment at two sites). Of these 46 participants, 22 were randomly assigned to SMA-CC sequence and 24 to CC-SMA sequence. Three withdrawals occurred during the first study period (one during SMA and two during CC). Forty-three participants completed the study and were analysed. One participant was excluded from CHO metrics analyses and number of manual correction boluses per period since a high proportion of boluses were given directly via the pump and not via the meal bolus section on the app, precluding estimation of these metrics.

**Fig. 2:**
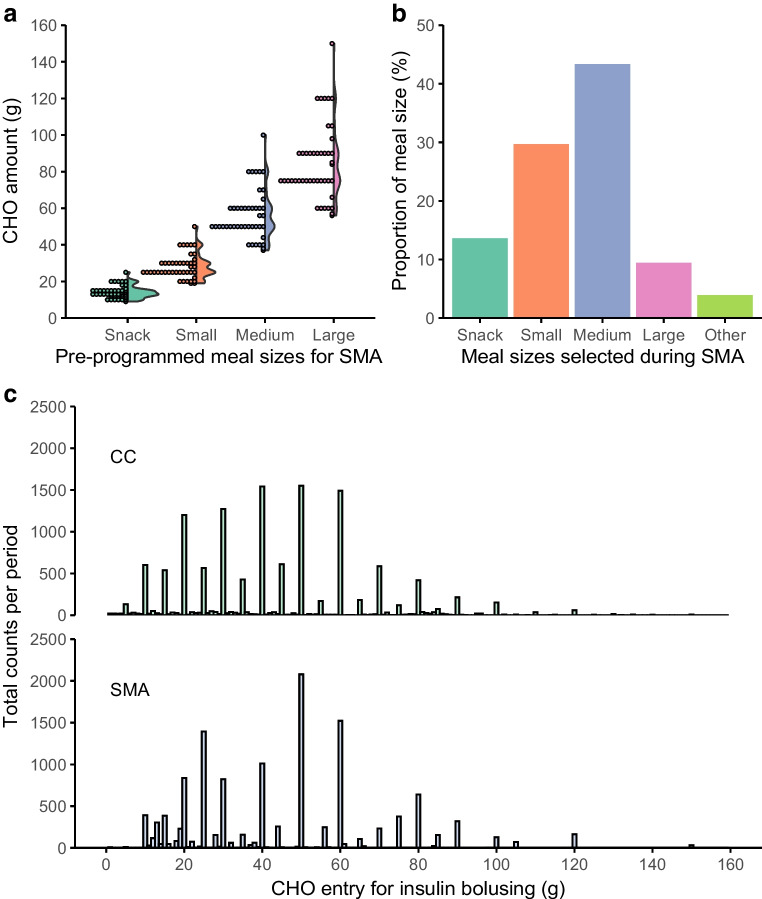
(**a**, **b**) Meal-size-specific pre-programmed carbohydrate (CHO) content (**a**) and percentage of each standard CHO meal size selected with SMA (**b**). (**c**) Distribution of entered CHO contents with CC and SMA across participants. ‘Other’, deviation from pre-set CHO amount. Data from 43 participants

Baseline characteristics of participants are displayed in Table 1. Participants were aged 16.4±2.1 years (range 12–20), all of white ethnicity, and 42% were female. Socioeconomic status was not specifically assessed. Similar sex distribution was found in other studies performed in youth and young adults with type 1 diabetes [20, 21]. Treatment modality at baseline was multiple daily injections (MDI) in 77%, sensor-augmented pump therapy in 9%, and HCL pump therapy in 14% of the participants. All MDI-treated participants, except for one person, were using continuous or intermittently scanned glucose monitoring. Baseline HbA_1c_ was 58.0±10.6 mmol/mol (7.5±1.0%) (range 33.3–89.1 mmol/mol [5.2–10.3%]). The mean absolute CC error, assessed using the food picture quiz, was 23.4±7.4 g (51.3±35.9%) with systematic underestimation (bias −16.3±14.0 g). According to 3-day food records, self-reported mean CHO intake was 29.3±23.7 g for breakfast, 55.4±22.4 g for lunch, 58.8±20.3 g for dinner and 16.7±19.9 g for snacks. All participants used faster acting insulin aspart (Fiasp) during the entire study period except for one person who switched to insulin aspart (Novorapid) 2 weeks after study initiation. Meal-specific standard CHO contents during SMA and CC are illustrated in Fig. 2a, b and ESM Table 4. CIR settings and changes to CIR settings are reported in ESM Table 5.

**Table 1:** Characteristics of the trial participants at baseline

Characteristic	Total (*n*=43)	SMA-CC sequence (*n*=21)	CC-SMA sequence (*n*=22)
Age, years			
Mean	16.4±2.1	16.6±1.9	16.3±2.3
Range	12–20	13–20	12–20
Sex, *n* (%)			
Female	18 (41.9)	11 (52.4)	7 (31.8)
Male	25 (58.1)	10 (47.6)	15 (68.2)
Height, cm			
Mean	170.5±9.8	170.2±8.1	170.8±11.3
Range	145.1–192.6	155.0–186.6	145.1–192.6
Weight, kg			
Mean	66.4±10.9	69.0±8.0	64.0±12.8
Range	36.3–96.9	54.7–87.5	36.3–96.9
BMI, kg/m^2^			
Mean	22.8±3.3	23.9±3.1	21.8±3.2
Range	17.2–30.3	18.6–30.3	17.2–30.0
BMI *z* score^a^			
Mean	0.5±1.0	0.8±0.8	0.2±1.1
Range	−2.1 to 2.3	−1.0 to 2.3	−2.1 to 2.3
HbA_1c_, mmol/mol			
Mean	58.0±10.6	60.4±11.0	55.7±9.9
Range	33.3–89.1	46.4–89.1	33.3–69.4
HbA_1c_, %			
Mean	7.5±1.0	7.7±1.0	7.2±0.9
Range	5.2–10.3	6.4–10.3	5.2–8.5
Duration of diabetes, years			
Mean	7.9±4.8	7.6±4.8	8.1±5.1
Range	1–16	1–16	1–16
Total insulin dose, U/day			
Mean	54.8±14.0	56.4±15.6	53.2±12.6
Range	30–95	30–95	30–74
Total insulin dose, U kg^−1^ day^−1^			
Mean	0.8±0.2	0.8±0.2	0.8±0.2
Range	0.5–1.3	0.5–1.2	0.5–1.3
Treatment at recruitment, *n* (%)			
MDI	33 (76.7)	14 (66.7)	19 (86.4)
with glucose sensor	32 (97.0)	14 (100.0)	18 (94.7)
HCL system	6 (14.0)	3 (14.3)	3 (13.6)
Sensor-augmented pump	4 (9.3)	4 (19.0)	0 (0)
History of severe hypoglycaemia, *n* (%)	8 (18.6)	4 (19.0)	4 (18.2)
Participants with HbA_1c_ <53 mmol/mol (7%), *n* (%)	15 (34.9)	6 (28.6)	9 (40.9)

Data are *n* (%) or mean±SD

^a^*z* score adjusted for age and sex based on the 2000 CDC growth chart

**Fig. 3:**
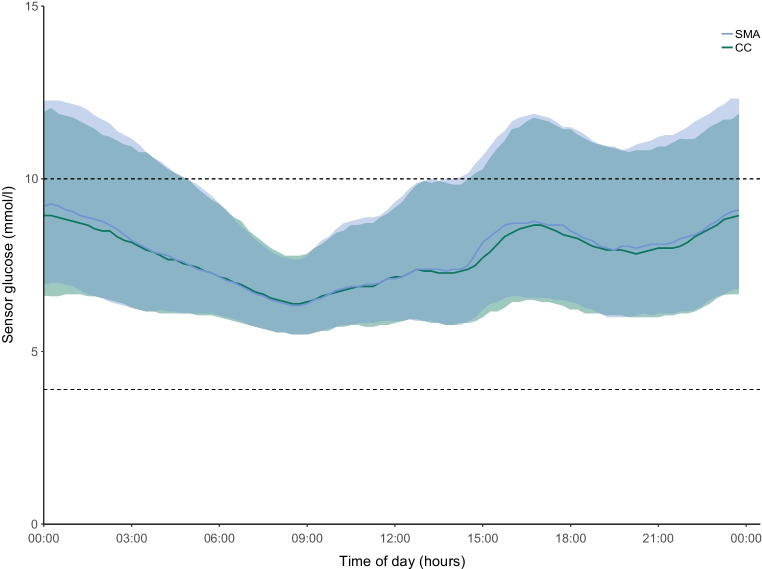
Median (IQR) sensor glucose profile during the SMA period (blue line and shaded area; *n*=43) and the CC period (green line and shaded area; *n*=43). Blue and green shaded areas represent IQR for each period. Black horizontal dashed lines represent lower and upper limits of the glucose target range of 3.9–10.0 mmol/l

Primary and key secondary endpoints are summarised in Table 2 (glucose endpoints separated for intervention sequences are described in ESM Table 6). Figure 3 illustrates the 24 h glucose profile. Postprandial glucose changes of declared meals containing >25 g of CHO during breakfast (05:00–11:00), lunch (11:00–17:00) and dinner (17:00–23:00) as well as mean peak postprandial glucose within 180 min following main meals are shown in ESM Fig. 3 and ESM Table 7.

**Table 2:** Comparison of the primary endpoint and key secondary endpoints for SMA and CC

	SMA (*n*=43)	CC (*n*=43)	Estimated difference or ratio (95% CI)	*p* value^a^
Glucose metrics				
Percentage of time with glucose concentration in range				
3.9–10.0 mmol/l (%)	69.9±12.4	70.7±13.0	−0.6 (−2.4, 1.1)^b^	0.48
<3.9 mmol/l (%)	1.8 [1.2–2.2]	1.9 [1.6–2.5]	0.9 (0.8, 1.0)^c^	0.12
<3.0 mmol/l (%)	0.3 [0.1–0.5]	0.3 [0.2–0.4]	0.8 (0.6, 1.0)^c^	0.046
>10.0 mmol/l (%)	28.2±12.6	27.2±13.4	0.8 (−1.0, 2.6)	0.35
>13.9 mmol/l (%)	8.4 [4.0–14.4]	7.5 [4.5–12.4]	1.1 (1.0, 1.2)^c^	0.19
Mean glucose, mmol/l	8.7±1.2	8.7±1.4	0.1 (−0.1, 0.3)	0.49
CV of glucose, %	38.7±6.1	38.2±6.2	−0.6 (−0.2, 1.3)	0.14
SD of glucose, mmol/l	3.4±0.9	3.4±1.0	0.07 (−0.04, 1.18)	0.20
GMI, %	7.1±0.5	7.0±0.6	0.3 (−0.6, 1.2)	0.49
Insulin delivery metrics				
TDD, U/day	54.0±14.7	51.7±12.1	2.4 (0.1, 4.6)	0.037
TDD, U kg^−1^ day^−1^	0.79±0.17	0.76±0.14	0.03 (−0.00, 0.06)	0.056
Daily bolus insulin dose, U/day	17.7±9.1	18.1±8.9	−0.3 (−1.4, 0.8)	0.58
Bolus percentage, %	33.0±14.0	35.0±14.1	−1.9 (−3.6, −0.3)	0.023
Daily basal insulin dose, U/day	36.4±13.0	33.6±10.9	2.7 (0.9, 4.5)	0.0041
Basal percentage, %	67.0±14.0	65.0±14.1	1.9 (0.3, 3.6)	0.023
Daily number of boluses, *n*	3.5±1.1	3.7±1.4	−0.1 (−0.4, 0.1)	0.21
Bolus dose, U	5.0±1.8	4.9±1.7	0.1 (−0.2, 0.4)	0.49
Number of manual correction boluses per period, *n*	2.0 [0.0–10.5]^d^	2.0 [0.0–12.8]^d^	0.5 (−0.5, 0.01)	0.95
CHO metrics				
Total amount of CHO entered per day, g/day	153.5±68.3^d^	155.7±62.8^d^	−0.5 (−9.1, 8.2)	0.91
Mean meal CHO content, g	44.8±14.5^d^	43.8±12.8^d^	0.9 (−1.6, 3.4)	0.47

Data are mean±SD or median [IQR]

Key glucose endpoints from intention-to-treat analysis separated for intervention sequences can be found in ESM Table 6

^a^*p* values are based on two-sided tests using generalised linear mixed-effect models

^b^Primary endpoint

^c^Non-normally distributed data are presented as ratio of SMA over CC, with 95% CI for the ratio; a value greater than unity indicates that the measurement was higher in SMA than in CC

^d^One participant was excluded from the analyses of the CHO metrics and number of manual correction boluses per period because a high proportion of boluses were given directly by the pump and not the meal bolus section on the app

The primary endpoint, which was the proportion of time sensor glucose was in the target range between 3.9 mmol/l and 10.0 mmol/l, was 69.9±12.4% with SMA and 70.7±13.0% with CC, with a mean difference of −0.6pp (95% CI −2.4, 1.1), demonstrating non-inferiority in the intention-to-treat analysis. The change in HbA_1c_ was similar between interventions (SMA −3.9±10.3 mmol/mol [−0.35±0.94pp], CC −3.4±10.6 mmol/mol [−0.31±0.97pp], *p*=0.59).

Time spent with glucose <3.9 mmol/l (median [IQR] 1.8 [1.2–2.2] vs 1.9 [1.6–2.5]%, *p*=0.12), >10.0 mmol/l (28.2±12.6 vs 27.2±13.4%, *p*=0.35) and >13.9 mmol/l (8.4 [4.0–14.4] vs 7.5 [4.5–12.4]%, *p*=0.19) was comparable in both study periods. Time with glucose <3.0 mmol/l was significantly lower with SMA (mean difference 0.8pp [95% CI 0.6, 1.0], *p*=0.046). Mean glucose (8.7±1.2 vs 8.7±1.4 mmol/l, *p*=0.49), CV of glucose (38.7±6.1 vs 38.2±6.2%, *p*=0.14), SD of glucose (3.4±0.9 vs 3.4±1.0 mmol/l, *p*=0.20) and GMI (7.1±0.5 vs 7.0±0.6%, *p*=0.49) did not significantly differ between study periods.

The number of events with glucose <3.9 mmol/l lasting for at least 15 min and extended hypoglycaemia (glucose <3.9 mmol/l lasting at least 120 min) did not significantly differ between periods (mean difference −0.1 events [95% CI −0.2, 0.005], *p*=0.062 and −0.5 events [95% CI −1.1, 0.1], *p*=0.12, respectively). Extended hyperglycaemic events (>13.9 mmol/l for ≥120 min) were significantly more frequent with SMA vs CC (mean difference 0.1 events [95% CI 0.02, 0.2], *p*=0.019). Frequency of events with glucose <3.0 mmol/l lasting for at least 15 min was significantly lower with SMA vs CC (mean difference −0.2 events [95% CI −0.3, 0.002], *p*=0.047). Further details are reported in ESM Table 7.

Table 2 illustrates insulin and CHO metrics (endpoints separated for intervention sequences can be found in ESM Table 8). Number of carbohydrate entries for hypoglycaemia correction (median [IQR] SMA 14.0 [3.0–42.5] vs CC 15.0 [3.5–36.0], *p*=0.71) did not significantly differ between periods. TDD was significantly higher during SMA vs CC (54.0±14.7 U/day vs 51.7±12.1 U/day, mean difference 2.4 U/day [95% CI 0.1, 4.6, *p*=0.037]), which was attributable to higher automated insulin delivery (36.4±13.0 U/day vs 33.6±10.9 U/day, *p*=0.0041). Bolus insulin dose was 17.7±9.1 U/day with SMA and 18.1±8.9 U/day with CC, *p*=0.58. The distribution of selected/entered meal CHO contents with SMA and CC is illustrated in Fig. 2c.

Bolus frequency and doses, both for meals and corrections, did not significantly differ between periods. Likewise, total daily CHO entry and meal CHO content were similar and food frequency questionnaires suggested comparable energy intake and macronutrient distribution (ESM Table 9).

In the SMA-CC sequence group, body weight increased from 69.0±8.0 kg at baseline to 70.4±8.4 kg at study end. In the CC-SMA sequence group, baseline weight was 64.0±12.8 kg and increased to 66.3±12.6 kg at study end. The increase in body weight (+1.1±2.1 vs +0.8±2.3 kg for SMA and CC, respectively, *p*=0.42) and BMI (kg/m^2^) (+0.3±0.8 vs +0.1±0.8 kg/m^2^, *p*=0.19) did not significantly differ between periods. The BMI *z* scores remained stable from start to end of the study (0.003±0.3 SMA-CC group vs 0.06±0.2 CC-SMA group, *p*=0.67, ESM Table 10).

Continuous glucose monitoring (CGM) availability (96.2±3.4% in SMA and 95.9±4.1% in CC, *p*=0.56) and time in HCL operation (90.3±6.4% vs 89.7±8.4%, *p*=0.45) were similar in both study periods.

User experience, as reflected in the INSPIRE (Insulin delivery Systems: Perceptions, Ideas, Reflections and Expectations) scores, did not significantly differ between study periods (SMA 66.4±14.3, CC 67.1±16.4, *p*=0.74). Experienced device deficiencies (mainly pump occlusion alarms) are detailed in ESM Table 11.

Safety endpoints are shown in ESM Table 12. Three SAEs occurred during the study: two episodes of diabetic ketoacidosis (one in each study period) and one severe hypoglycaemia during CC. None of the SAEs were related to the treatment intervention. Fifteen adverse events were reported, of which ten were adverse device effects. The number of safety events did not differ between periods (all *p*>0.40).

## Discussion

This two-centre, open-label, crossover RCT investigated the glycaemic efficacy of HCL insulin therapy using the mylife CamAPS FX system with SMA vs CC in youth and young adults with type 1 diabetes. Our findings confirmed the study hypothesis that SMA was non-inferior to CC with a mean treatment difference in time with glucose in target range of −0.6pp (95% CI −2.4, 1.1). Non-inferiority was also supported by the per-protocol analysis (ESM Table 2). While small differences in other glucometrics and insulin delivery (approximately 4.5% higher dose with SMA vs CC) were observed, our study does not indicate any clear disadvantage of SMA compared with CC.

Two other RCTs have recently contrasted SMA with CC during HCL insulin therapy in people with type 1 diabetes [22, 23], albeit with different HCL systems. In contrast to our findings, both studies could not confirm non-inferiority of SMA. Petrovski et al [23] addressed the research question with the MiniMed 780G HCL system in 34 adolescents (aged 12–18 years) with type 1 diabetes in a two-arm parallel RCT over 3 months. Time in target range with SMA was 6.8pp lower than with CC and time spent at glucose levels >13.9 mmol/l was significantly higher, whereas other endpoints did not significantly differ. Unlike in our study, participants (71% prior MDI users) were scheduled to use the HCL system for their usual care and those with CHO estimation errors >20% were excluded. Active treatment optimisation was performed repeatedly by the study team. Since the MiniMed 780G HCL currently does not offer an SMA interface, participants most likely had to manually enter their assigned standard CHO meal size into the bolus calculator, which is in contrast with the selection of meal symbols in our study. The frequency of meal bolus events was significantly lower in the SMA vs CC group (3.7 vs 5.1, respectively), while meal bolus frequency in our study did not differ between interventions. The second RCT conducted by Haidar et al [22] compared SMA vs CC during HCL insulin therapy in 30 adults with type 1 diabetes (mean age 44 years) over a short period of 3 weeks in a crossover design. The HCL system consisted of a non-commercial research device and, unlike in our study, SMA settings (<30 g, 30–60 g, 60–90 g and >90 g CHO) were identical for all participants. Time in range was 70.5% with SMA and 74.1% with CC (mean difference of −3.6pp [95% CI −6.5, 0.6]) [22]. With a non-inferiority margin of 4pp, the trial did not demonstrate non-inferiority of SMA compared with CC. In line with our findings, SMA resulted in higher automated insulin delivery and hence higher TDD compared with CC. In Europe, apart from the CamAPS FX algorithm, the Diabeloop Generation 1 (DBLG1) system is currently the only CE-marked alternative allowing for SMA via selection of pre-set meal sizes. A retrospective real-world study analysing data of 1958 DBLG1 users aged ≥18 years contrasted glucose control during days with SMA (85,554 days) vs CC (81,173 days) for meal bolus delivery [24]. Although time spent in target range was significantly lower with SMA than CC (69.7 vs 70.7%, *p*<0.01), the difference of <1% was not deemed clinically meaningful and other glucometrics were comparable. The iLet Bionic Pancreas system, which is currently only available in the USA [25], offers a distinct SMA approach. In contrast to the CamAPS FX system, the iLet Bionic Pancreas does allow the user to select meal sizes in the absence of pre-set CHO meal sizes. The user announces the type of the meal (breakfast, lunch or dinner) and indicates whether the amount of consumed carbohydrates is ‘usual for me’, ‘more’, or ‘less’ for that meal type, allowing the system to address meal-specific insulin requirements. Neither entry of CHO in grams nor programming of CIR are possible in this system.

In light of the current evidence, we interpret our study findings of non-inferiority of SMA vs CC in the context of the performance of an adaptive control algorithm, which continuously modulates insulin delivery using a physiological model of the glucoregulatory system [26]. The learning of the algorithm considers meal bolus information to adapt post-meal insulin delivery, particularly when meals are not adequately covered by user-initiated bolus administration. Thus, in the scenario of meal CHO portions falling below actual amounts, the algorithm will compensate for the missing insulin. Evidence suggests that people with type 1 diabetes tend to underestimate CHO quantities, especially with larger amounts of CHO [9] and systematic underestimation of CHO was also found in our study population, as suggested by the negative bias in the CHO quiz and the pattern of CHO entries. The lack of evidence of higher hypoglycaemia exposure with SMA compared with CC (time with glucose <3.9 mmol/l was low in both study periods) supports the safety of SMA use with the current HCL system, in particular when pre-set CHO quantities are on the lower side of actually consumed quantities and tailored to individual eating habits. Contrary to our expectations, we observed lower time in hypoglycaemia <3.0 mmol/l with SMA vs CC, but hypoglycaemia burden was low during both study periods. The number of extended hyperglycaemic events was significantly higher during SMA than CC, but the difference was marginal.

Despite the higher insulin doses with SMA vs CC, we did not observe any differences in weight changes between the periods. Nonetheless, a statistically significant weight gain was noticeable in both study periods, which we interpret mainly in the context of physiological growth, supported by the stability of BMI *z* scores. Whether corrective insulin doses of an adaptive control algorithm can lead to mild but constant supraphysiological insulin exposure remains speculative at this stage. In view of the rising obesity rates among people with type 1 diabetes [27] and some study findings supporting unfavourable eating patterns with automated insulin delivery (e.g. slippage into increased snacking), attention to this matter may be required [28]. In the present study, we assessed dietary habits using a food frequency questionnaire and could not observe any significant differences during the course of the study. However, the used methodology does not qualify for in-depth insights into potential changes in eating behaviour. As the relationship between automated insulin delivery systems, eating behaviour and metabolic health in people with type 1 diabetes is incompletely understood, there is a clear need for further investigations, especially in people with overweight or obesity. Finally, alleviation of CC and optimisation of metabolic health may also involve the use of adjunctive treatments (e.g. glucagon-like peptide-1 [GLP-1] receptor agonists, pramlintide and sodium–glucose cotransporter [SGLT-2] inhibitors), as supported by previous research [29–31]. However, associated side effects and safety concerns may limit their use in growing children and teenagers.

User satisfaction did not increase with SMA in the present study. It is important to note that the use of the SMA in the HCL system did not reduce the number of interactions with the app (e.g. similar number of buttons that needed to be pressed for bolus delivery). We therefore believe that simpler user interfaces have the potential to improve the usability of HCL systems in future. Experienced technical issues (pump occlusion alarms) may also have affected participants’ overall satisfaction, especially in prior MDI users who accounted for 77% of the study population and may have lower tolerance thresholds. Still, time in auto mode was similar to previously published studies in youth and young adults with type 1 diabetes [19, 32]. Further improvement of technical robustness and usability of systems are an important requirement to support HCL adoption in this target group. In our study, the number of participants reaching the recommended HbA_1c_ target of <53 mmol/mol (7.0%) after study completion nearly doubled compared with baseline. Observed time in range in our study was comparable with findings of other trials in similar populations [19, 33, 34] with glucose levels trending upwards towards the end of the 6 month period, highlighting the challenge of sustaining treatment engagement in this population. Similarly, engagement challenges with prolonged HCL use in young people were observed in previous research [35]. Sex-related disparities in study outcomes are of interest. Although the trial was not powered to test for sex-specific differences, sex-disaggregated analyses will be reported in a separate work.

Strengths of our study include the two-centre randomised crossover design, the sufficient study length allowing assessment of longer-term treatment effects and impact on HbA_1c_, as well as the lack of remote monitoring and active treatment optimisation, representing real-world use. We were also able to answer a clinically relevant research question with potential impact on future design of HCL systems and education guidelines. While more research is needed on the impact of different control algorithms and the profile and preference of the end user, it is probable that recommendations for meal management during HCL use will be revised, in particular with increasing research on the use of artificial intelligence-enhanced meal anticipation and pharmacological innovations [36]. We acknowledge several limitations. Applicability of our findings to the wider type 1 diabetes population may be limited given the focus on adolescents and young adults with the majority being HCL-naive. It is conceivable that people with poor baseline glucose control or CC skills may have benefitted from the SMA strategy. However, CHO estimation skills are difficult to evaluate, and the consistency of the error may be more relevant than its magnitude. Furthermore, the adopted study design does not allow for conclusions regarding the timing of bolus delivery, which has relevant impact on glucose control [8]. Lastly, the implementation of the SMA did not lead to reduced treatment effort, calling for more user-friendly strategies (e.g. home screen button press) to increase treatment satisfaction.

In conclusion, SMA results in non-inferior glycaemic control but a modest increase in TDD compared with CC in adolescents and young adults with type 1 diabetes using the mylife CamAPS FX HCL system. Our findings therefore suggest that simplifying meal management during HCL therapy may be a viable strategy to reduce the burden of CC without worsening glucose control. However, this conclusion is dependent on the characteristics and specific needs of the user as well as the type of automated insulin dosing algorithm of the HCL system.

## Supplementary Information

Below is the link to the electronic supplementary material.

ESM (PDF 604 KB)

## Data Availability

De-identified subject level dataset will be made available on a case-by-case basis on reasonable request to the corresponding author for research purposes 6 months after publication.

## Abbreviations

CC: Carbohydrate counting
CHO: Carbohydrates
CIR: Carbohydrate to insulin ratio
DBLG1: Diabeloop Generation 1
GMI: Glucose management indicator
HCL: Hybrid closed-loop
MDI: Multiple daily injections
pp: Percentage points
SAE: Serious adverse event
SMA: Simplified meal announcement
TDD: Total daily insulin dose
