# Supplementary material for: Simplified meal announcement study (SMASH) using hybrid closed-loop insulin delivery in youth and young adults with type 1 diabetes: a randomised controlled two-centre crossover trial
Source: Diabetologia. 2024 Nov 19;68(2):295–307. doi: 10.1007/s00125-024-06319-w (PMC11732900; doi:10.1007/s00125-024-06319-w)
Supplement: Supplementary file 1 — ESM (PDF 604 KB) [file 125_2024_6319_MOESM1_ESM.pdf]

Electronic supplementary material

Electronic supplementary figures

ESM Fig. 1. Study flow Page 2

ESM Fig. 2. CONSORT flow diagram Page 3

ESM Fig. 3. Postprandial median changes in glucose levels Page 4-5

Electronic supplementary tables

ESM Table 1. Eligibility criteria Page 6

ESM Table 2. Glucose endpoints as per-protocol analysis Page 7

ESM Table 3. Protocol deviations from the per-protocol analysis Page 8

ESM Table 4. Pre-programmed app settings, simplified meal announcement (SMA) and carbohydrate counting (CC) Page 9

ESM Table 5. Changes in carbohydrate-insulin-ratio Page 10

ESM Table 6. Glucose endpoints as per intention-to-treat separated for intervention sequences Page 11

ESM Table 7. Mean peak postprandial glucose endpoints and number of hypoglycaemic and hyperglycaemic events Page 12

ESM Table 8. Comparison of insulin delivery and app-derived meal carbohydrate (CHO) metrics during study periods separated for intervention sequences Page 13

ESM Table 9. Average daily energy intake and macronutrient distribution Page 14

ESM Table 10. Clinical characteristics of participants during study separated for intervention sequences Page 15

ESM Table 11. Device deficiencies Page 16

ESM Table 12. Safety endpoints Page 17

ESM Fig. 1. Study flow

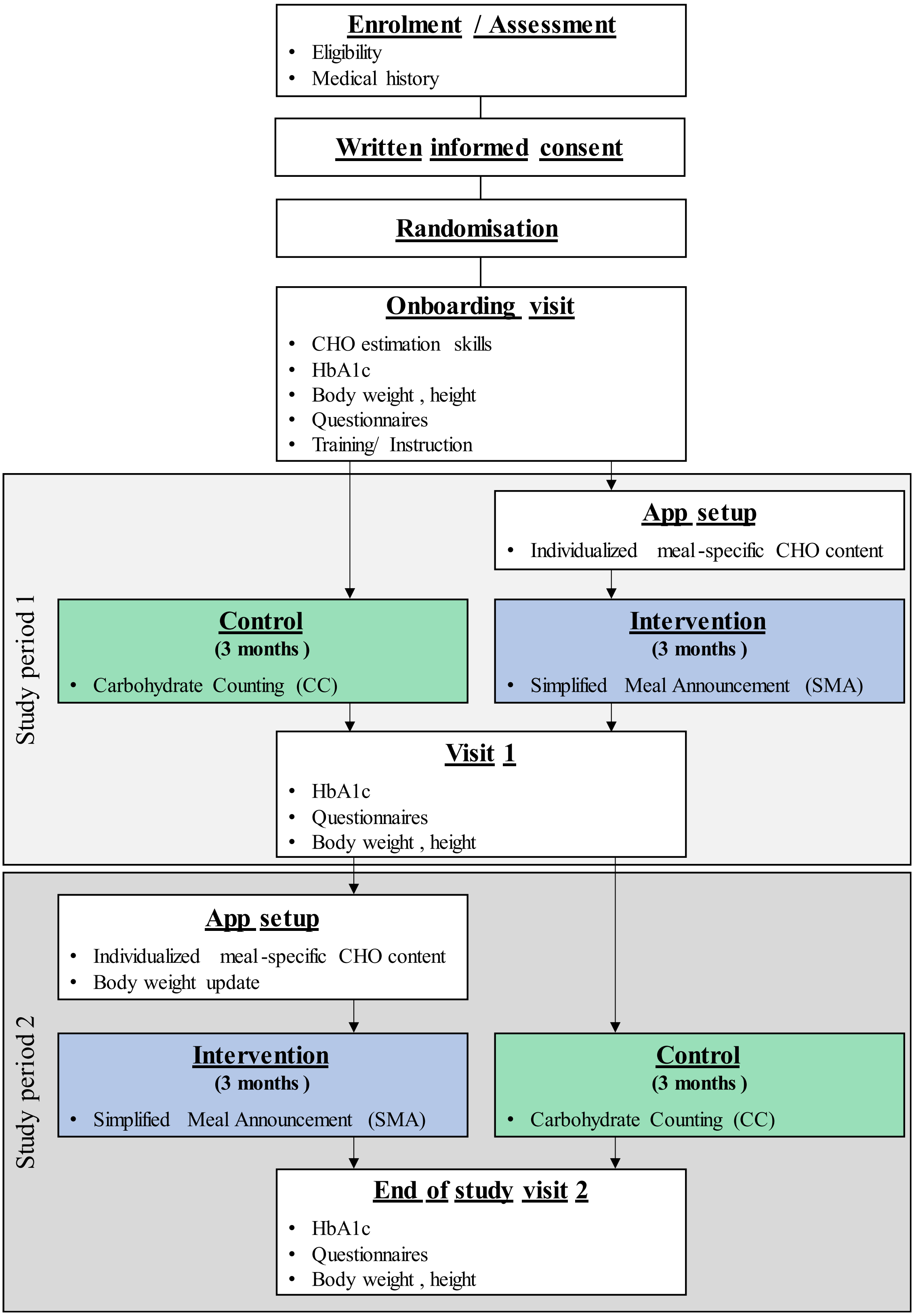

ESM Fig. 1: Study flow. SMA = simplified meal announcement, CC = carbohydrate counting, HbA1c = glycated haemoglobin, CHO = carbohydrates.

ESM Fig. 2. CONSORT flow diagram

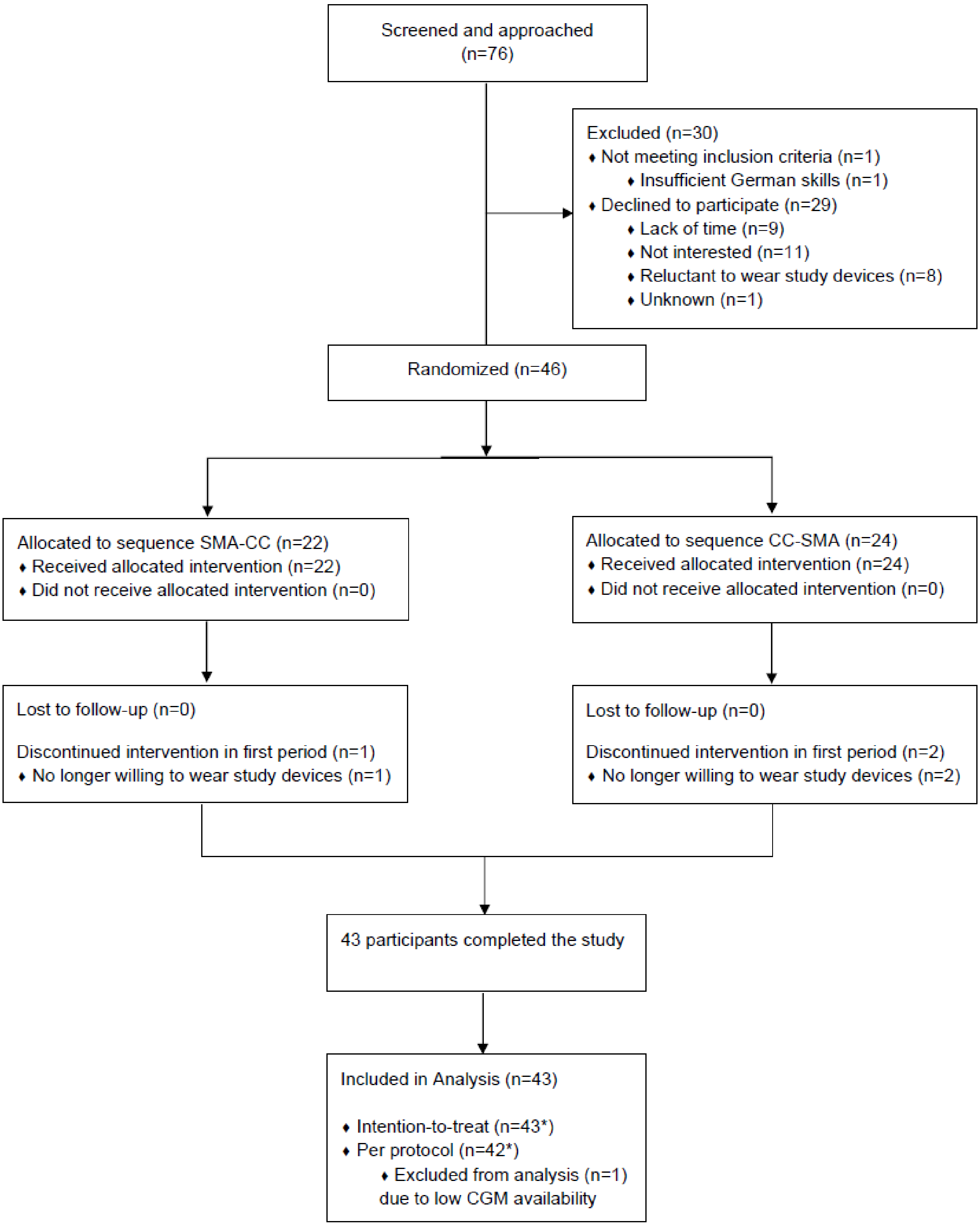

ESM Fig. 2: CONSORT flow diagram. One participant in the SMA-CC sequence discontinued intervention after 65 days, one participant from CC-SMA sequence discontinued after 53 days and the other one after 19 days. *One participant was excluded from the analyses of CHO metrics and number of manual correction boluses per period because a high proportion of boluses were given directly by the pump and not the meal bolus section on the app.

ESM Fig. 3. Postprandial median changes in glucose levels

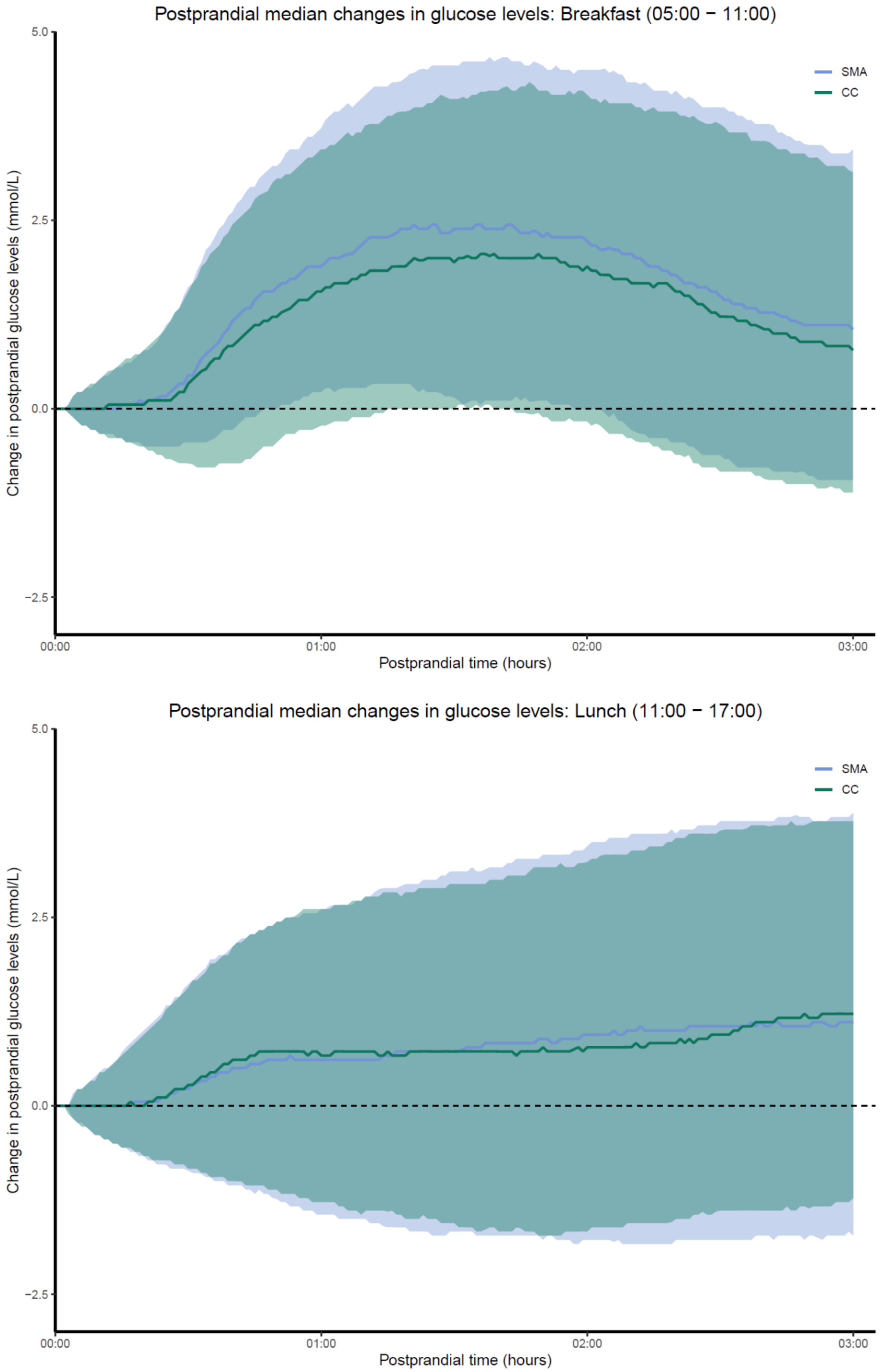

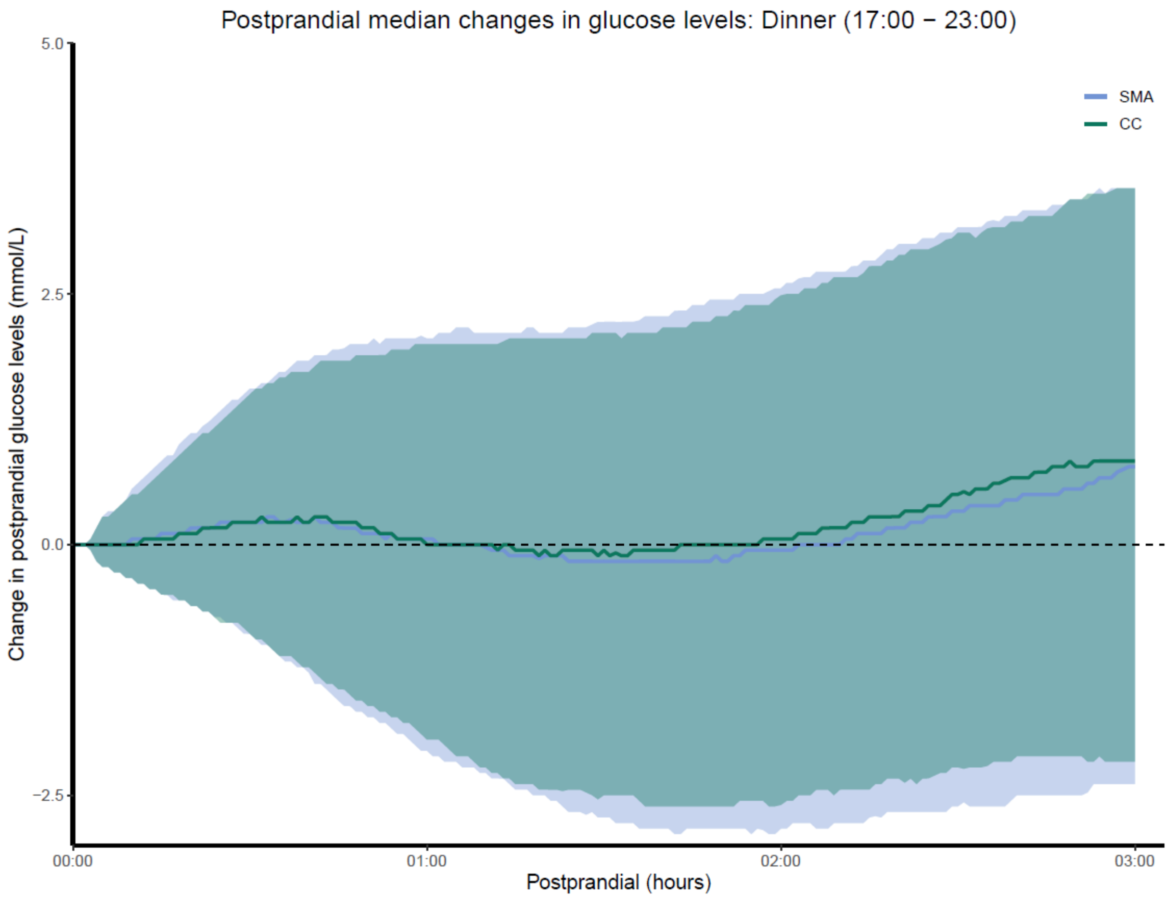

ESM Fig. 3: Postprandial median changes in glucose levels after meal inputs >25g for breakfast (05:00-11:00), lunch (11:00-17:00) and dinner (17:00-23:00). Shaded area demonstrates interquartile ranges. SMA= simplified meal announcement, CC= carbohydrate counting.

ESM Table 1. Eligibility criteria

Participants fulfilling all of the following inclusion criteria were eligible for the study:

- Written informed consent
- Type 1 diabetes as defined by World Health Organisation for at least 6 months
- Age between 12 and 20 years (inclusive)
- Proficient use of continuous glucose monitoring or flash glucose monitoring for at least 5 days in the past
- Glycated haemoglobin ≤12%
- The participant is willing to wear closed-loop devices
- The participant is willing to follow study specific instructions
- Negative urine-pregnancy test in sexually active female participants of childbearing potential at screening-visit

The presence of any one of the following exclusion criteria will lead to the exclusion of the subject

- Any physical or psychological disease or condition likely to interfere with the normal conduct of the study and interpretation of the study results
- Known or suspected allergy against insulin
- Participant is pregnant or breast feeding or planning pregnancy within next 6.5 months
- Severe visual impairment
- Severe hearing impairment
- Lack of reliable telephone facility for contact
- Concomitant participation in another trial that interferes with the normal conduct of the study and interpretation of the study results
- Participant not proficient in German

ESM Table 2. Glucose endpoints as per-protocol analysis

	Simplified meal announcement (n=42[a])	Carbohydrate counting (n=42 [a])	Group difference (95% CI)	p-value
**Percentage of time with glucose concentration in range**				
3.9-10.0mmol/L (%)	71.3±11.9	71.8±11.7	-0.2 [-2.0; 1.6]	0.81
<3.9mmol/L (%)	1.9 [1.3; 2.2]	2.1 [0.0; 0.0]	0.88 [0.78; 1.00][b]	0.055
<3.0mmol/L (%)	0.30 [0.13; 0.36]	0.36 [0.21; 0.42]	0.96 [0.92; 0.995][b]	0.028
>10.0mmol/L (%)	26.8±12.0	26.1±12.0	0.4 [-1.4; 2.3]	0.62
>13.9mmol/L (%)	9.1 [3.7; 12.9]	8.7 [4.2; 11.9]	0.03 [-0.08; 0.14][b]	0.58
**Mean glucose (mmol/L)**	8.7±1.2	8.7±1.4	-1.2 [-2.9; 0.5]	0.55
**CV of glucose (%)**	38.2±6.1	37.9±6.1	4.2 [-2.0; 10.4]	0.60
**SD of glucose (mmol/L)**	3.3±0.9	3.3±0.8	0.2 [-0.4; 0.7]	0.48
**GMI (%)**	7.0±0.5	7.0±0.5	0.02 [-0.55; 0.10]	0.55

Eligibility for the per-protocol analysis was continuous glucose monitoring (CGM) availability above 80% in both periods. Additionally, with SMA, only days with exclusive use of the standard CHO sizes were included in the analysis and for both periods, only days with at least one meal bolus were considered. Also in both periods, only days without the use of other meal functions such as “add meal” were analysed.

In the per-protocol analysis, deviations from protocol lead to exclusion of 16.1% [5.9; 24.5] of days during SMA (deviations from pre-set CHO meal sizes or days without any manual bolus delivery) and 0.5% [0; 3.4] of days during CC (days without any manual bolus delivery). A detailed description of protocol deviations can be found in ESM Table 3. Data are mean±SD or median [IQR]. CV = Coefficient of variation, SD = Standard deviation, GMI = Glucose Management Indicator.

[a] One participant was excluded from per-protocol analysis due to CGM availability of 75.9% in the second study period.

[b] Non-normally distributed data are presented as ratio of SMA over CC, with 95% CI for the ratio; a value greater than unity indicates that the measurement was higher in SMA than in CC.

ESM Table 3. Protocol deviations concerning the per-protocol analysis

	Simplified meal announcement (n=43)	Carbohydrate counting (n=43)	Group difference (95% CI)	p-value
**Days without exclusive use of the meal size icon selection during SMA period (days)**	12 [4; 19.5]	N.A.	N.A.	N.A.
**Days without any manual bolus delivery (days)**	1 [0; 2.5]	1 [0; 3.5]	0.02 [-0.3; 0.4]	0.90
**Days with the use of the add meal function other than for hypoglycaemia correction (days)**	0 [0; 0.5]	0 [0; 0.5]	-0.03 [-0.9; 0.8]	0.93

Data are median [IQR]. SMA= Simplified meal announcement, N.A. = not applicable.

ESM Table 4. Pre-programmed app settings, simplified meal announcement (SMA) and carbohydrate counting (CC)

	Simplified meal announcement (n=43)	Carbohydrate counting (n=43)
**Meal-specific standard CHO contents**		
Snack (g CHO)		
Mean	14.2±3.5	N.A.
Range	9-25	N.A.
Small (g CHO)		
Mean	28.2±6.9	N.A.
Range	19-50	N.A.
Medium (g CHO)		
Mean	56.3±13.8	N.A.
Range	37-100	N.A.
Large (g CHO)		
Mean	84.5±20.7	N.A.
Range	56-150	N.A.
**Carbohydrate to insulin ratio (g/U)**		
Mean	9.2±2.4	9.1±2.3
Range	5.0-15.8	5.0-15.0

Data are mean±SD or range. CHO = carbohydrates, N.A. = not applicable, SD = standard deviation

ESM Table 5. Changes in carbohydrate-insulin-ratio

	Simplified meal announcement (n=43)	Carbohydrate counting (n=43)	Number of changes (n=54)	Number of participants (n=43)
**Number of participants with (n)**				
0 changes per period	29 (67.4%)	30 (69.8%)		
≥1 change per period	14 (32.6%)	13 (30.2%)		
≥2 changes per period	6 (14.0%)	9 (20.9%)		
≥3 changes per period	4 (9.3%)	4 (9.3%)		
≥4 changes per period	3 (7.0%)	1 (2.3%)		
**Number of changes per period (n)**				
changes in first period			46 (85.2%)	
changes in second period			8 (14.8%)	
**Number of participants with (n)**				
no changes in both periods				21 (48.8%)
changes in one period				17 (39.5%)
changes in both periods				5 (11.6%)

Data are n (%).
2 out of 43 participants decided to use meal-time-specific CIR throughout the study, while the remainder used a single 24h carbohydrate-to-insulin ratio (CIR) throughout the study. There were no statistically significant differences in the frequency of CIR changes between SMA and CC (SMA 27 changes, CC 27 changes, p=n.s.). Mean signed changes in CIR did not differ between periods (SMA +12.4±17.1%, CC +11.0±19.4%, p=n.s.) and were predominantly implemented in the first study period.

ESM Table 6. Glucose endpoints as per intention-to-treat separated for intervention sequences

	SMA-CC sequence (n=21)		CC-SMA sequence (n=22)	
	**Simplified meal announcement (period 1)**	**Carbohydrate counting (period 2)**	**Carbohydrate counting (period 1)**	**Simplified meal announcement (period 2)**
**Percentage of time with glucose concentration in range**				
3.9-10.0mmol/L (%)	70.3±10.8	65.0±13.2	76.2±10.4	69.8±14.1
<3.9mmol/L (%)	2.0 [1.2; 2.3]	1.9 [1.5; 2.4]	2.0 [1.7; 2.5]	1.7 [1.2; 2.2]
<3.0mmol/L (%)	0.27 [0.11; 0.45]	0.39 [0.21; 0.42]	0.27 [0.21; 0.35]	0.27 [0.20; 0.44]
>10.0mmol/L (%)	28.0±11.1	33.1±13.5	21.5±10.7	28.3±14.1
>13.9mmol/L (%)	8.4 [4.7; 11.6]	9.0 [7.5; 15.7]	5.7 [2.7; 7.8]	8.3 [3.2; 15.1]
**Mean glucose (mmol/L)**	8.7±1.1	9.2±1.5	8.1±1.1	8.8±1.4
**CV of glucose (%)**	39.1±4.9	39.8±4.6	36.6±7.2	38.4±7.3
**SD of glucose (mmol/L)**	3.4±0.8	3.7±0.9	3.0±0.9	3.5±1.1
**GMI (%)**	7.1±0.5	7.3±0.6	6.8±0.5	7.1±0.6

Data are mean±SD or median [IQR]. SMA= Simplified meal announcement, CC= carbohydrate counting, CV = Coefficient of variation, SD = Standard deviation, GMI = Glucose Management Indicator.

ESM Table 7. Mean peak postprandial glucose endpoints and number of hypoglycaemic and hyperglycaemic events

	Simplified meal announcement (n=43)	Carbohydrate counting (n=43)	Group difference (95% CI)	p-value
**Mean peak postprandial glucose (mmol/L) within 180min following main meals** [a]				
00:00 to 23:59	12.5±2.0	12.3±2.1	0.3 [-0.005; 0.6]	0.054
05:00 to 10:59	12.2±2.3	11.9±2.1	0.3 [-0.4; 1.0]	0.34
11:00 to 16:59	12.5±2.1	12.1±1.9	0.3 [-0.1; 0.7]	0.12
17:00 to 22:59	12.8±2.1	12.6±2.5	0.3 [-0.03; 0.6]	0.073
**Number of events with glucose <3.9mmol/L for ≥15min per period (n)**				
00:00 to 23:59	67.0±30.6	74.2±36.3	-0.1 [-0.2; 0.005]	0.062
00:00 to 05:59	14.9±11.0	14.2±10.3	0.03 [-0.1; 0.2]	0.69
06:00 to 23:59	52.4±24.0	59.7±31.2	-0.1 [-0.2; -0.01]	0.033
**Number of events with glucose <3.9mmol/L lasting at least 120min per period (n)**				
00:00 to 23:59	0 [0; 1]	0 [0; 1.5]	-0.5 [-1.1; 0.1]	0.12
00:00 to 05:59	0 [0; 0]	0 [0; 1]	-0.5 [-1.2; 0.3]	0.21
06:00 to 23:59	0 [0; 0]	0 [0; 0]	-0.5 [-1.4; 0.4]	0.23
**Number of events with glucose <3.0mmol/l for ≥15min per period (n)**				
00:00 to 23:59	10 [7; 16]	12 [9; 18]	-0.2 [-0.3; -0.002]	0.047
00:00 to 05:59	2 [1; 5]	4 [1.25; 6]	-0.1 [-0.3; 0.2]	0.63
06:00 to 23:59	8 [4.5; 11]	9 [5.5; 12]	-0.2 [-0.4; -0.01]	0.041
**Number of events with glucose >13.9mmol/l lasting at least 120min per period (n)**				
00:00 to 23:59	27.0 [10.5; 46.5]	24.0 [13.0; 41.5]	0.1 [0.02; 0.2]	0.019
00:00 to 05:59	8.0 [3; 13]	6.5 [3; 10.75]	0.1 [-0.1; 0.3]	0.47
06:00 to 23:59	17.0 [8.0; 36.5]	17.0 [9.0; 33.5]	0.2 [0.04; 0.3]	0.0096
**Number of participants with ≥70% time with glucose in the target range (3.9-10.0mmol/L)**	24 (55.8%)	26 (60.5%)		0.53

Intention-to-treat analysis. Data are mean±SD, median [IQR] or n (%). SD = standard deviation.

[a] defined as carbohydrate amounts >25g entered into the mylife CamAPS FX app by the participants.

ESM Table 8. Comparison of insulin delivery and app-derived meal carbohydrate (CHO) metrics during study periods separated for intervention sequences

	SMA-CC sequence (n=21)		CC-SMA sequence (n=22)	
	**Simplified meal announcement (period 1)**	**Carbohydrate counting (period 2)**	**Carbohydrate counting (period 1)**	**Simplified meal announcement (period 2)**
**Insulin delivery metrics**				
Total daily insulin dose (U/d)	57.3±17.1	52.1±13.1	51.4±11.5	50.9±11.4
Total daily insulin dose (U/kg/d)	0.80±0.18	0.73±0.12	0.79±0.15	0.78±0.17
Daily bolus insulin dose (U/d)	20.3±9.6	16.4±7.7	19.7±9.7	15.2± 8.1
Bolus percentage (%)	35.2±12.2	31.2±12.3	38.6±15.0	30.8±15.5
Daily basal insulin dose (U/d)	37.1±12.5	35.7±10.3	31.7±11.3	35.7±13.7
Basal percentage (%)	64.8±12.2	68.7±12.3	61.4±15.0	69.2±15.5
Daily number of boluses (n)	3.8±1.0	3.4± 1.1	3.9±1.6	3.2±1.1
Bolus dose (U)	5.3±1.8	4.7±1.6	5.1±1.7	4.8±1.9
Number of manual correction boluses per period (n)	2.5 [0.0; 15.3][a]	4.0 [0.0;.16.0][a]	1.0 [0.0; 6.8]	2.0 [0.3;.4.8]
**Carbohydrate metrics**				
Total amount of CHO entered per day (g/d)	170.5±73.4[a]	145.9±69.7[a]	164.6±56.0	138.1±60.9
Average meal CHO content (g)	45.4±13.0[a]	42.8±12.5[a]	44.6±13.3	44.1±16.1

Data are mean±SD or median [IQR]. SMA= Simplified meal announcement, CC= carbohydrate counting, CHO = Carbohydrates.

[a] One participant was excluded from the analyses of the CHO metrics and number of manual correction boluses per period because a high proportion of boluses were given directly by the pump and not the meal bolus section on the app.

ESM Table 9. Average daily energy intake and macronutrient distribution

	Simplified meal announcement (n=42) [a]	Carbohydrate counting (n=42) [a]	Group difference (95% CI)	p-value [b]
**Daily energy intake (kcal/24h)**	1554.7±849.5	1759.9±980.9	0.9 (0.8; 1.0) [c]	0.18
Percentage calories from carbohydrates (%)	47.0±11.1	46.3±10.0	0.7 (-1.7; 3.1)	0.57
Percentage calories from fibers (%)	2.4±0.8	2.4±0.8	-0.0 (-0.2; 0.2)	0.83
Percentage calories from fats (%)	32.8±9.5	34.1±9.7	1.0 (0.9; 1.1) [c]	0.47
Percentage calories from proteins (%)	17.8±6.6	17.2±6.2	1.0 (0.9; 1.1) [c]	0.48

Distribution of daily energy intake for simplified meal announcement (SMA) and carbohydrate counting (CC) assessed using a food frequency questionnaire. Data are mean±SD. Cal = calories, SD = standard deviation.

[a] One participant was excluded from the analysis due to incomplete questionnaires

[b] p-values were obtained using two-tailed tests assessing non-equivalence.

[c] Non-normally distributed data are presented as ratio of SMA over CC, with 95% CI for the ratio; a value greater than unity indicates that the measurement was higher in SMA than in CC.

ESM Table 10. Clinical characteristics of participants during study separated for intervention sequences

	SMA-CC sequence (n=21)		CC-SMA sequence (n=22)	
**Characteristic**	**Simplified meal announcement (period 1)**	**Carbohydrate counting (period 2)**	**Carbohydrate counting (period 1)**	**Simplified meal announcement (period 2)**
Height (cm)	170.4±8.2	171.0±8.4	171.4±11.0	172.1±10.6
Weight (kg)	70.8±8.5	70.4±8.4	65.9±13.2	66.3±12.6
BMI (kg/m^2)	24.5±3.5	24.2±3.2	22.3±3.6	22.3±3.5
BMI Z-Score [a]	0.9±0.9	0.8±0.8	0.3±1.2	0.2±1.1
Glycated haemoglobin (%; mmol/mol)	6.6±0.7; 48.8±7.9	7.0±1.1; 53.0±11.7	6.3±0.7; 45.1±7.4	6.6±0.9; 48.6±9.8
Participants with glycated haemoglobin <53mmol/mol (7%) (n)	19 (90.5%)	14 (66.7%)	19 (86.4%)	14 (63.6%)

Data are mean±SD or n (%). SMA= simplified meal announcement, CC= carbohydrate counting.

[a] Z-Score adjusted for age and sex based on the 2000 CDC growth chart.

ESM Table 11. Device deficiencies

Event	Simplified meal announcement (n=44)	Carbohydrate counting (n=45)
**Number of recorded device deficiencies per period (n)**	607 (49.3%)	623 (50.7%)
**Insulin pump**	589 (97.0%)	613 (98.4%)
mylife CamAPS FX app connectivity	2 (0.3%)	0 (0%)
Electronic errors [a]	12 (2.0%)	23 (3.7%)
Occlusion alarms	575 (94.7%)	590 (94.7%)
**Glucose sensor**	12 (2.0%)	6 (1.0%)
mylife CamAPS FX app connectivity	8 (1.3%)	1 (0.2%)
Device malfunction	2 (0.3%)	4 (0.6%)
Discrepancy with blood glucose levels	2 (0.3%)	1 (0.2%)
**User errors**	6 (1.0%)	4 (0.6%)

Data are n (%). Device malfunction consisted of early sensor termination, impossibility to insert sensor due to technical issues and sensor errors. User error consisted of destruction of devices e.g. during physical activity and incautious behaviour.

[a] Pump replacement due to electronic errors was carried out in 11 participants

ESM Table 12. Safety endpoints

	Simplified meal announcement (n=44)	Carbohydrate counting (n=45)
**Number of adverse events**	6	9
Number of participants (%)	4 (9.1%)	8 (17.8%)
**Number of serious adverse events**	1	2
Severe hypoglycaemia	0	1
Diabetic ketoacidosis	1	1
**Number of adverse events** [a]	5	7
Adverse device effects	4	6
Adverse events unrelated to study intervention	1	1

Data are n (%).

[a] Adverse device effects consisted of hyperglycaemia without ketosis, haematoma and pain at sensor insertion site, skin irritation to CGM adhesive, infusion-set related skin irritation and discrepancy from sensor to blood glucose levels leading to hyperglycaemia without ketosis. Adverse events unrelated to the study consisted of hay fever attack and abdominal pain.
